# Stimulation of hair growth by Tianma Gouteng decoction: Identifying mechanisms based on chemical analysis, systems biology approach, and experimental evaluation

**DOI:** 10.3389/fphar.2022.1073392

**Published:** 2022-12-16

**Authors:** Yanyan Zhang, Shiqian Zhang, Yunluan Long, Wuji Wang, Fanpan Du, Jingjie Li, Feng Jin, Zheng Li

**Affiliations:** ^1^ Key Laboratory of Basic Pharmacology of Ministry of Education and Joint International Research Laboratory of Ethnomedicine of Ministry of Education, Zunyi Medical University, Zunyi, Guizhou, China; ^2^ Key Laboratory of Basic Pharmacology of Guizhou Province, Zunyi Medical University, Zunyi, Guizhou, China; ^3^ Department of Pharmacology, School of Pharmacy, Zunyi Medical University, Zunyi, Guizhou, China

**Keywords:** Tianma Gouteng decoction, molecular docking, alopecia, hair growth, network pharmacology, Wnt/β-catenin

## Abstract

Hair serves important physiological functions, including temperature regulation and scalp protection. However, excessive shedding not only impacts these functions but can also significantly affect mental health and quality of life. Tianma Gouteng decoction (TGD) is a traditional Chinese medicine used for the treatment of various conditions, including hair loss. However, the associated mechanism underlying its anti-alopecia effect remains unknown. Therefore, this study aims to elucidate these mechanisms by employing systematic biology approaches, as well as *in vitro* and *in vivo* experimental validation. The chemical constituents of Tianma Gouteng decoction were identified using UHPLC-MS/MS, from which 39 potential bioactive components were screened, while an additional 131 putative Tianma Gouteng decoction beneficial components were extracted from the Traditional Chinese Medicine Database and Analysis Platform (TCMSP) database. We then applied a dual-dimensional network pharmacology approach to analyze the data, followed by validation studies combining molecular docking techniques with *in vivo* and *in vitro* experiments. From the 39 bioactive components, including quercetin, luteolin, fisetin, wogonin, oroxylin A, boldine, tetrahydroalstonine, and galangin A, 782 corresponding targets were identified. In particular, GSK3β and β-catenin exhibited strong binding activity with the bioactive compounds. Hence, construction of a bioactive component-target network revealed that the mechanism underlying the anti-alopecia mechanism of Tianma Gouteng decoction primarily involved the Wnt/β-catenin signaling pathway. Moreover, C57BL/6J mice exhibited measurable improvements in hair follicle regeneration following treatment with Tianma Gouteng decoction. Additionally, β-catenin and p-GSK3β levels were upregulated, while GSK3β was downregulated in Tianma Gouteng decoction-treated animals and dermal papilla cells compared to control group. These *in vivo* and *in vitro* outcomes validated the targets and pathways predicted in the network pharmacology analysis of Tianma Gouteng decoction. This study provides a systematic analysis approach to identify the underlying anti-alopecia mechanisms of Tianma Gouteng decoction, further providing theoretical support for clinical assessment of Tianma Gouteng decoction.

## 1 Introduction

Alopecia, also called hair loss, is caused by hair follicle atrophy that can lead to abnormal alopecia (Dhariwala and Ravikumar, 2019; Baek et al., 2022). Approximately 40% of women worldwide suffer from hair loss, while the proportion of hair loss in males is much higher at 85% (Zito and Raggio, 2022). Although hair loss is not life-threatening, excessive shedding can negatively impact mental health and quality of life, leading to depression and other health conditions (Aukerman and Jafferany, 2022; Macbeth et al., 2022). Hair growth is cyclical and includes three consecutive phases: anagen (growth phase), catagen (regression phase), and telogen (resting phase) (Paus and Foitzik, 2004; Lin et al., 2022). Regulation of the hair growth cycle is a common strategy for treating hair loss. In fact, two drugs, minoxidil and finasteride, have been approved by the United States Food and Drug Administration for this purpose. However, due to the associated adverse events, patient compliance for these drugs is poor. For example, finasteride can significantly reduce libido, cause erectile dysfunction, and abnormal sexual function (Arca et al., 2004; Sato and Takeda, 2012; Dou et al., 2022). Moreover, the pathological mechanism of hair loss is complex, making effective treatment with a single compound challenging. Hence, a need exists for the development of more effective and less toxic therapeutic options.

Traditional Chinese Medicine (TCM) has a long history of clinical application for myriad conditions and diseases, representing a unique health resource. In particular, various TCM preparations based on plant monomers have exhibited excellent results in promoting hair growth (Kang et al., 2012; Kang et al., 2019; Her et al., 2022), encouraging a more in-depth investigation of such treatments. This approach has the advantage of providing multi-component, multi-target synergistic effects while with low toxicity. Tianma Guoteng decoction (TGD) is a TCM preparation described in the book “New Meaning of Miscellaneous Diseases Treatment”. TGD contains 11 ingredients, namely: *Gastrodia elata Blume* [Orchidaceae; Gastrodiae Rhizoma, GR 9 g], *Uncaria rhynchophylla (Miq.) Miq. Ex Havil.* [Rubiaceae; Uncariae Ramulus cum Uncis, UR 12 g], *Haliotis diversicolor Reeve* [Haliotidae; Haliotidis Concha, HC 18 g], *Gardenia jasminoides J. Ellis* [Rubiaceae; Gardeniae Fructus, GF 9 g], *Scutellaria baicalensis Georgi* [Lamiaceae; Scutellariae Radix, SR 9 g], *Eucommia ulmoides Oliv.* [Eucommiaceae; Eucommiae Cortex, EC 9 g], *Leonurus japonicus Houtt.* [Lamiaceae; Leonuri Herba, LH 9 g], *Taxillus chinensis (DC.) Danser* [Loranthaceae; Taxilli Herba, TH 9 g], *Reynoutria multiflora (Thunb.) Moldenke* [Polygonaceae; Polygoni Multiflori Caulis, PMC 9 g], *Faria cocos (Schw.) Wolf* [Polyporaceae; Poria, PA 9 g], and *Cyathula officinalis K.C. Kuan* [Amaranthaceae; Cyathulae Radix, CR 12 g]. Pharmacological studies have demonstrated that TGD exhibits anti-oxidant, anti-apoptotic, and neuroprotective (Liu et al., 2015; Xian et al., 2016) effects. Moreover, it has proven effective in the treatment of hypertension, Parkinson’s disease, and hair loss (Zhang et al., 2003; Tsai et al., 2014; Liu et al., 2015; Deng et al., 2022). In fact, many components of TGD have exhibited beneficial therapeutic effects on hair loss. For example, baicalin (the active ingredient of HQ) effectively treats hair loss *via* promotion of hair follicle development through activation of the Wnt/β-catenin pathway (Xing et al., 2018). Meanwhile, quercetin (the active ingredient in DZ) is used to prevent and treat hair loss by promoting the expression of growth factors (Wikramanayake et al., 2012; Kim et al., 2020). Hence, TGD has potential as an effective complementary and alternative medicine option for hair loss treatment, however, a complete list of its bioactive components, and the associated therapeutic mechanisms, remain to be characterized.

**FIGURE 1:**
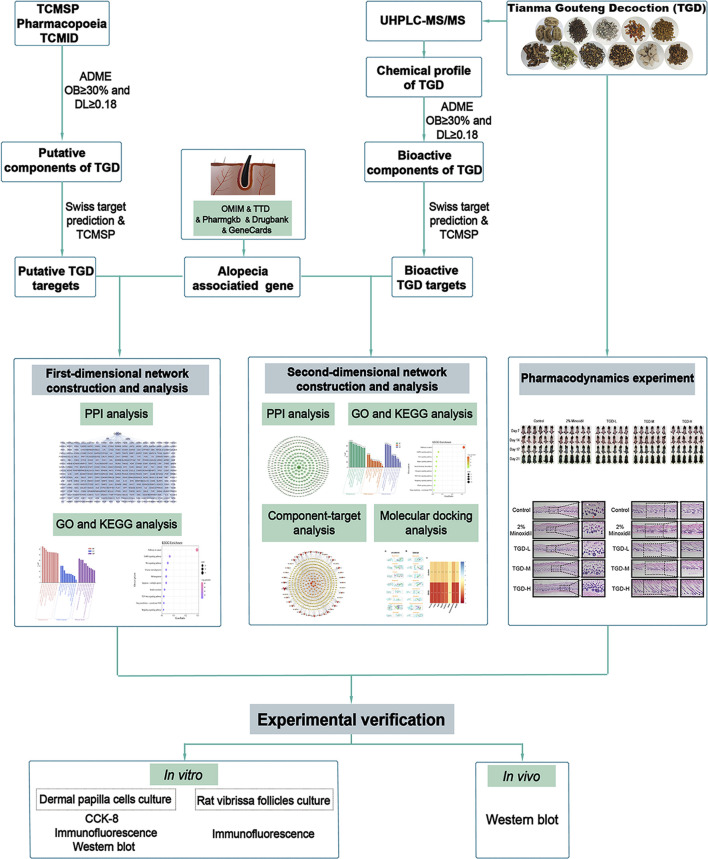
The technical strategy of the current study.

TCM formulas are characterized by their multi-component composition that provides multi-target synergistic actions. However, the use of traditional pharmacological methods to study the biological mechanisms of these medicines is limited. In contrast, network pharmacology is a new strategy that combines computer technology and systems biology to explore associated pathways (Zhou et al., 2020; Wei et al., 2022). Compared with traditional approaches, this technique can decipher the complex relationships between biological systems, medicine, and diseases in a multidimensional manner from a network perspective (Zhang et al., 2019). Herein, we describe a dual-dimensional pharmacological network utilizing the active components of TGD as selected from the Traditional Chinese Medicine Database and Analysis Platform (TCMSP) database and detected by ultra-high performance liquid chromatography-tandem mass spectrometry (UHPLC-MS/MS). We propose potential bioactive compounds involved in the anti-alopecia mechanism using these multiple perspectives, and subsequently validating our data with molecular docking, *in vivo* experiments, and *in vitro* analyses (Figure 1).

## 2 Material and methods

### 2.1 Chemicals and reagents

HPLC grade methanol, acetonitrile, and acetic acid were obtained from ANPEL Laboratory Technologies (Shanghai, China) and analytical grade ethanol from Titan Scientific (Shanghai, China). GR (No. 201101), UR (No. 220418), HC (No. 200701), GF (No. 20030101), SR (No. 2010061), EC (No. 200801), LH (No. 200701), TH (No. 200825), PMC (No. 200602), PA (No. 200818), and CR (No. 200501) were obtained from Tongji Tang Pharmaceutical (Guizhou, China). Insulin, hydrocortisone, and dihydrotestosterone were sourced from Solarbio Technology (Beijing, China). Sigma-Aldrich (St. Louis, MO, United States) supplied testosterone. A Cell Counting Kit-8 (CCK-8) was ordered from Dojin Laboratories (Kunamoto, Japan). Dulbecco’s modified eagle medium (DMEM), William’s E Medium, 0.25% trypsin, penicillin, streptomycin, phosphate-buffered saline (PBS), and fetal bovine serum (FBS) were obtained from Gibco (Auckland, New Zealand). Antibodies against β-catenin (8480S) and glycogen synthase kinase-3 beta (GSK3β; 12,456) were sourced from Cell Signaling Technology (Boston, United States). Signalway Antibody (College Park, United States) supplied the anti-p-GSK3β antibody (11,002). Anti-LEF1 antibody (ab137872) and goat anti-rabbit IgG H&L (Alexa Fluor 488) (ab150077) were purchased from United Kingdom Abcam-Abbo Trading (Shanghai, China). Goat anti-rabbit IgG-horseradish peroxidase (HRP)-conjugated secondary antibodies (abs20040) were obtained from Absin Biotechnology (Shanghai, China).

### 2.2 Preparation of TGD

The quality of the raw TGD extract was strictly controlled in accordance with the Chinese Pharmacopoeia. We combined ingredients at a mass ratio of 9:12:18:9:9:9:9:9:9:9:12 for GR:UR:HC:GF:SR:EC:LH:TH:PMC:PA:CR. The mixture was soaked overnight with 90% ethanol at a ratio of 1:20 and exposed to ultrasonic extraction for 30 min the next day. Following filtration, a first filtrate was concentrated using rotary evaporation. The filter residue was collected and the above steps were repeated to obtain a second filtrate. The process was altered in the production of a third filtrate, which was obtained using aqueous extraction and subsequently concentrated. Subsequently, all three filtrates were combined into a lyophilized powder, precipitated by alcohol to remove impurities, and concentrated. The lyophilized powder was stored at 4°C without light exposure.

### 2.3 Chromatographic and mass spectrometric conditions

The lyophilized TGD powder of TGD (sourced from 2.2) was re-dissolved in 30% acetonitrile, and supernatants were obtained for UHPLC-MS/MS analysis using 1.4 × 10^4^ g centrifugation for 15 min. UPLC separation was performed by a Waters HSS T3 chromatographic column (100 mm × 2.1 mm, 1.8 μm) at 40 °C, with a mobile phase comprising 0.1% formic acid-water solution (A phase) and 0.1% formic acid-acetonitrile (B phase). The flow rate of mobile phase was set at 0.3 ml/min and injection volume was 2 µl. The optimal gradient elution program was performed: 0–1 min, 100% (A); 1 min–9 min, 5% (A)-95% (B); 9 min–15 min, 50% (A)-50% (B); 15.1 min–17 min, 100% (A). Auto-sampler tray temperature was maintained at 4°C throughout the analysis. The high-resolution mass spectrometry system was run in the positive and negative ionization modes of electrospray ionization (ESI), with mass range from *m/z* 70–17500 Da. MS parameter conditions were as follows: source temperature: 350°C; curtain gas (CUR): 25 psi; ion source gas1(Gas 1) and ion source gas2(Gas 2): 50 psi and 50 psi respectively; capillary voltage: -2800 V/3000 V; sheath gas pressure: 40 psi; auxiliary gas: 10 psi.

For data acquisition, we used the Progenesis QI software (Waters Corporation, Milford, United States). After peak identification, retention time correction, peak alignment, the components of TGD were preliminarily determined according to the *m/z* value and the retention time compared with the database (http://www.hmdb.ca/ and https://metlin.scripps.edu/). When necessary, MS spectra was compared with standards to further identify the components. In addition, the measured masses of components were all within a mass deviation of 10 ppm, which making the data more reliable.

### 2.4 Network pharmacology and molecular docking analysis

#### 2.4.1 Screening of TGD compounds and their protein targets

To facilitate interpretation of the complex mechanism underlying the effects of TGD on hair loss, a dual-dimensional network was created using TGD components from different sources (one from databases, the other identified by UHPLC-MS/MS). First, the putative beneficial compounds of TGD were identified using oral bioavailability (OB) and drug-likeness (DL) as filter criteria in the TCMSP (http://tcmspw.com/tcmsp.php), TCM Taiwan Systems Pharmacology (TCM Database@Taiwan, http://tcm.cmu.edu.tw), and Traditional Chinese Medicines Integrated Database (TCMID, http://www.megabionet.org/tcmid/). The screening criteria were OB ≥ 30% and DL ≥ 0.18. Moreover, the TGD putative beneficial compounds were supplemented by the Pharmacopoeia of the People’s Republic of China and published literature. Second, UHPLC-MS/MS identified potential bioactive compounds of TGD. The same screening criteria described above were applied. Finally, the targets corresponding to the components were assessed using the TCMSP database and Swiss Target Prediction platform (http://www.swisstargetprediction.ch). The latter selects targets based on a probability greater than zero. We imported the target names into the UniProt database (https://www.uniprot.org/) and converted them into official gene names. Targets of compounds were limited to human physiology.

#### 2.4.2 Prediction of alopecia therapeutic targets

We conducted five databases searches to identify alopecia-related genes, with the search keywords “hair growth” and “hair loss”. The databases included Online Mendelian Inheritance in Man (http://omim.org/), Therapeutic Target Database (https://db.idrblab.org/ttd/), the Pharmacogenomics Knowledgebase (https://www.pharmgkb.org/), Drugbank (https://go.drugbank.com/), and Genecards (https://www.genecards.org/). Potential alopecia-related genes identified from the five databases were combined into a list and duplicate values were removed to obtain the potential therapeutic targets for AGA.

#### 2.4.3 Protein-protein interaction construction and analysis

The list of overlapping targets was obtained by intersecting potential drug targets in TGD (described in 2.4.1) with presumed alopecia disease targets (described in 2.4.2). The resultant Venn diagram of overlapping targets was mapped using jvenn (http://jvenn.toulouse.inra.fr/app/example.html). Targets in common lists represented that were likely to be representative therapeutic targets for alopecia in TGD, we construction PPI (protein-protein interaction) network by importing overlapping targets to the String database (https://string-db.org/), with the targets were selected as “Homo sapiens species”. The results were constructed a target-target network using Cytoscape v3.9.1.

#### 2.4.4 Functional enrichment analysis of therapeutic targets for alopecia in TGD

To investigate the biological function of potential targets related to hair loss, we used Metascape (https://metascape.org/gp/index.html#/main/step1) to perform gene ontology (GO) and Kyoto Encyclopedia of Genes and Genomes (KEGG) pathway enrichment analysis of the mechanism and pathway associated with TGD anti-alopecia effects. The GO analysis encompassed biological processes (BP), molecular functions (MF), and cellular components (CC). We considered *p* < 0.01 as statistically significant for the functional gene classes.

#### 2.4.5 Construction and analysis of bioactive component-target network

To decipher the complex relationships between bioactive components of TGD and therapeutic targets of alopecia, a list of key components (that have been identified by UHPLC-MS/MS and screened based on criteria in described in 2.4.1.) and overlap targets (bioactive compounds and disease) was submitted to Cytoscape v3.9.1. The Cytoscape v3.9.1 was used to perform the bioactive component-target network. The degree value identified the importance of network nodes in the network; that is, higher degree values represent more significant roles. The top eight components, based on the degree value analysis, were screened as the core bioactive components.

#### 2.4.6 Visualization and validation of molecular docking

The top eight core TGD components identified from the bioactive component-target network were included in molecular docking simulations with GSK3β and β-catenin (which function in the Wnt/β-catenin signaling pathway). The Mol2 formats of the bioactive components were obtained from TCMSP database, and the PBD format of the protein targets were saved from Protein Data Bank database (http://www1.rcsb.org/), then optimized by energy minimization utilizing AutoDock Tools v1.5 to remove excess structure and water molecules and add non-polar hydrogen and partial charges. After ligand and receptor processing, AutoDock Vina v1.1.2 was applied for molecular docking, and the optimal complex conformation was plotted using PyMOL v2.3.4.

### 2.5 Experimental verification of bioinformatics results

#### 2.5.1 Experimental animals

Six-week-old female C57BL/6J mice (18–20 g) and seven-week-old male Sprague-Dawley (SD) rats (260–300 g) were obtained from Sipeifu Biotechnology (Beijing, China). Mice were raised in an animal facility approved by the Key Laboratory of Basic Pharmacology, Zunyi Medical University, under a 12-h light-dark cycle. The cages were kept pathogen-free at a room temperature of 21°C–23°C and 48–52% humidity. Mice had free access to food and water. The animal study protocols were approved by the Laboratory Animal Welfare & Ethics Committee of Zunyi Medical University (ZMU21-2105–102) and were carried out in accordance with Chinese animal welfare guidelines.

#### 2.5.2 Treatment of mouse models

Six-week-old female C57BL/6J mice were fed for 7 days to adapt to conditions. Thereafter, the dorsal skin of each mouse was shaved to induce the telogen phase of depilation, a procedure previously reported for seven-week-old mice (Muller-Rover et al., 2001). This allows hair follicles to develop synchronously in the subsequent growth phase. The following day, mice were randomly divided into five groups (*n* = 6 per group): 1) control (75% ethanol in medium), 2) 2% minoxidil, 3) TGD-L (low TGD dose, 40 mg/kg/d), 4) TGD-M (medium TGD dose, 200 mg/kg/d), 5) TGD-H (high TGD dose, 400 mg/kg/d). The TGD doses were set based on previous studies (Jang et al., 2016; Chung et al., 2017) and data (unpublished data) from a preliminary TGD study for hair growth. The dorsum of animals was topically treated each day with 200 μL for 14 days. Changes in dorsal skin color were observed and photographed on days 7, 14, 17, and 21. At the end of the study, mice were anesthetized by intraperitoneal injection of 2% pentobarbital (40 mg/kg) and sacrificed by cervical dislocation; dorsal skin samples were collected and stored at -80°C until further analysis.

#### 2.5.3 Histological assays

Murine skin tissues (obtained in Section 2.5.2) were fixed in 4% paraformaldehyde solution, dehydrated using an ethanol gradient (70, 75, 80, 95, and 100%), and embedded in paraffin wax. Sections with a thickness of 6 μm were obtained using an RM2235 microtome (Leica Microsystems GmbH, Weitzlar, Germany) and rehydrated using an ethanol gradient (100%, 95%, 85%, and 75%), followed by staining with hematoxylin and eosin (HE). The slices were washed with PBS, dehydrated using ethanol gradients (80%, 90%, and 100%), sealed with neutral resin, and observed under a light microscope (Olympus Corporation, Tokyo, Japan). Cross-sections were used to quantify the number and size of hair follicles, while longitudinal sections were used to assess the morphology and length of hair follicles.

#### 2.5.4 Isolation and culture of rat vibrissa follicles

Vibrissa follicles were removed from seven-week-old male SD rats (Zhou et al., 2019), with subsequent isolation of the follicles conducted as previously described (Philpott and Kealey, 2000). Briefly, SD rats were euthanized with carbon dioxide (CO_2_). The mystacial pads were removed from the upper lips followed by placing them in PBS containing 100 units/mL penicillin and 100 μg/ml streptomycin. The surrounding connective tissue was carefully removed under a stereomicroscope (Olympus Corporation), and hair follicles were separated from the tissue mass without damaging them. Isolated follicles were placed in a 24-well plate containing 500 μl William’s E Medium (supplemented with 2 mM l-glutamine, 10 μg/ml insulin, and 50 nM hydrocortisone) and cultured at 37°C in 5% CO_2_. The isolated follicles were divided into three groups: the control group and groups with rat vibrissa follicles treated with low or high TGD doses (100 ng/ml and 400 ng/ml, respectively). Culture medium was changed every 3 days, during which the shape and length of rat vibrissa follicles were recorded using a stereomicroscope (Olympus Corporation). After 12 days of continuous treatment, the samples were embedded using optimal cutting temperature (OCT) compound and stored at -80°C.

#### 2.5.5 Cell culture and proliferation assay of dermal papilla cells

Dermal papilla cells (DPCs) were isolated from the whiskers of SD rats using existing procedures (Jahoda and Oliver, 1981; Bak et al., 2018; Kang et al., 2022). Thereafter, the isolated DPCs were seeded in 96-well plates (5 × 10^3^ cells/well) and cultured for 48 h in DMEM (20% FBS, 100 units/mL penicillin, and 100 μg/ml streptomycin). Cells were then treated with either a control solvent (serum-free medium containing 0.1% dimethyl sulfoxide) or TGD at 100, 200, or 400 ng/ml for 24 h. Finally, 10 μl of CCK-8 solution was added for 1 h before measuring absorbance values at 450 nm using a microplate reader (Thermo Scientific).

#### 2.5.6 Immunofluorescent staining of DPCs and vibrissa follicles

DPCs (2 × 10^4^ cells/well) were inoculated in 24-well plates with 20% FBS for 48 h, using the experimental groups described in section 2.9. Following 24 h of treatment, DPCs were fixed with 4% paraformaldehyde for 20 min, permeabilized with 0.5% TritonTM X-100 for 10 min, and exposed to 20% goat serum albumin blocking agent for 30 min. Thereafter, the DPCs were incubated for 16 h with primary antibodies against β-catenin (1:80), GSK3β (1:500), p-GSK3β (1:200), and LEF1 (1:500) at 4°C. Next, DPCs were incubated with an Alexa Fluor 488 (1:1000)-conjugated secondary antibody for 1 h and nuclei were counterstained with DAPI. Rat vibrissa follicles embedded in OCT were blocked with goat serum albumin after fixing with absolute ethanol. Incubation with primary and secondary antibodies followed the processes described for DPCs. Fluorescent images were acquired using an orthomosaic fluorescence microscope (Olympus Corporation).

#### 2.5.7 Western blotting

Cellular proteins were extracted after 24 h of incubation with 100 ng/ml, 200 ng/ml, or 400 ng/ml TGD. Skin samples were collected from the -80°C refrigerator. Following step usually performed after samples extracted. Cellular and skin samples were treated, on ice, with a high efficiency lysis buffer and phenylmethylsulfonyl fluoride, and centrifuged at 12,000 rpm for 10 min. Protein content in the supernatant was determined with a BCA kit (Generay, Shanghai, China). Equal amounts of protein extracts were separated with an 8% SDS-PAGE gel and electrotransferred onto polyvinylidene fiuoride membranes. After applying a rapid blocking solution to the membranes for 20 min, proteins were incubated overnight with primary antibodies against β-catenin (1:1000), GSK3β (1:1000), and p-GSK3β (1:750) at 4°C. The following day, membranes were washed with TBS-0.5% Tween 20, incubated with an anti-rabbit IgG (1:5000) secondary antibody for 30 min, and developed with ECL luminescence reagent. Data were analyzed using Image Lab software.

#### 2.5.8 Statistical analysis

Data were evaluated using SPSS software, employing one-way analysis of variance (ANOVA) and least significant difference (LSD) or Dunnett’s T3 tests. Results were reported as the mean ± standard error of the mean (SEM), Differences were considered statistically significant if the *p*-value was <0.05.

## 3 Results

### 3.1 Potential therapeutic targets of alopecia-related

Using “hair loss” and “hair growth” as keywords, a total of 3938 alopecia-related targets were collected from five databases after removal of duplicates. (Supplementary Table S1).

### 3.2 First-dimensional network construction and analysis

Under the criterions of OB ≥ 30% and DL ≥ 18%, we constructed the first-dimensional network using 131 putative beneficial components of TGD (removing duplicate values) retrieved from database searches, and 573 corresponding protein targets of these putative beneficial components identified from TCMSP and Swiss Target Prediction (Supplementary Table S2).

**FIGURE 2:**
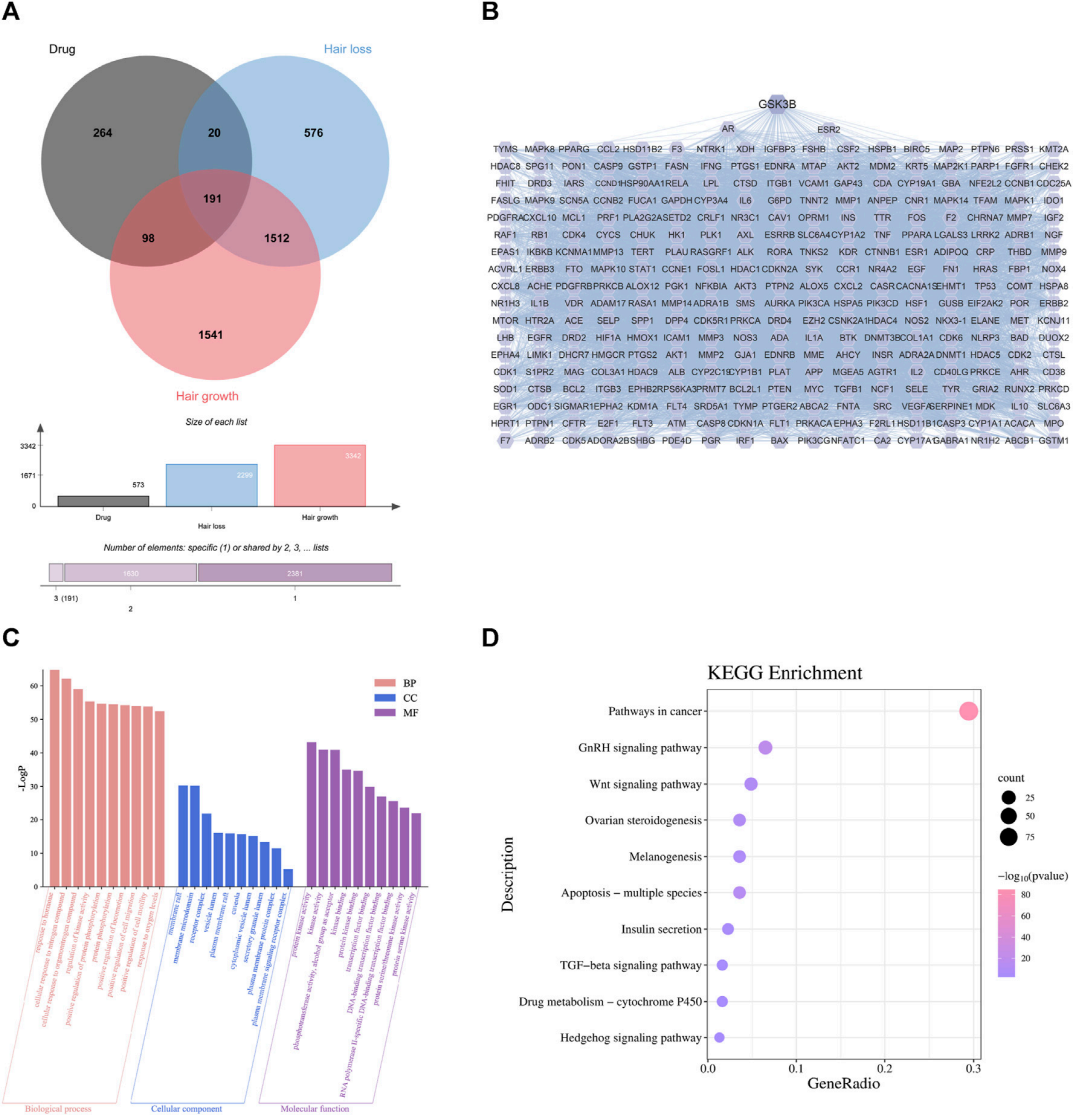
First-dimensional network constructed by putative beneficial components from TGD in alopecia. **(A)** Venn diagram of overlapping genes between putative beneficial components corresponding targets in TGD and alopecia targets. **(B)** PPI networks of common protein targets. **(C)** 10 pathways of BP, MF, and CC using GO enrichment analysis of intersection targets. **(D)** The bubble diagram of KEGG pathway enrichment analysis.

By comparing these targets with the 3938 alopecia-related targets in section 4.1, we identified 309 that occurred in both datasets as targets for TGD treatment of alopecia (Figure 2A, Supplementary Table S3). A total of 309 overlapping genes were imported into the String database to further investigate the relationships between these protein-protein interaction (PPI); PPI maps were constructed by transferring these genes to Cytoscape software (Figure 2B). The PPI analysis results included 309 nodes and 7934 edges. We ranked targets from high to low based on degree and used the average degree as a threshold to obtain 124 targets that were considered the most critical targets for TGD treatment of alopecia, including GSK3β. GSK3β was among the top 45 target genes in the PPI network.

To explore the underlying mechanism by which TGD promotes hair growth, GO and KEGG pathway enrichment analysis were performed on the 309 central targets while using Bioinformatics (http://www.bioinformatics.com.cn/) to display the top ten significantly enriched elements selected from the BP, CC, and MF categories (Figure 2C). We found that responses to hormones, positive regulation of cell migration and motility were involved in the biological process of hair growth. The KEGG enrichment further identified the involvement of multiple signaling pathways, including that of TGF-β, Hedgehog, and notably Wnt (Figure 2D). Further, there is previous evidence that the Wnt signaling pathway is involved in cell development and migration and plays a crucial role in the induction of hair follicle development (Rishikaysh et al., 2014; Steinhart and Angers, 2018).

### 3.3 Second-dimensional network construction and analysis

**FIGURE 3:**
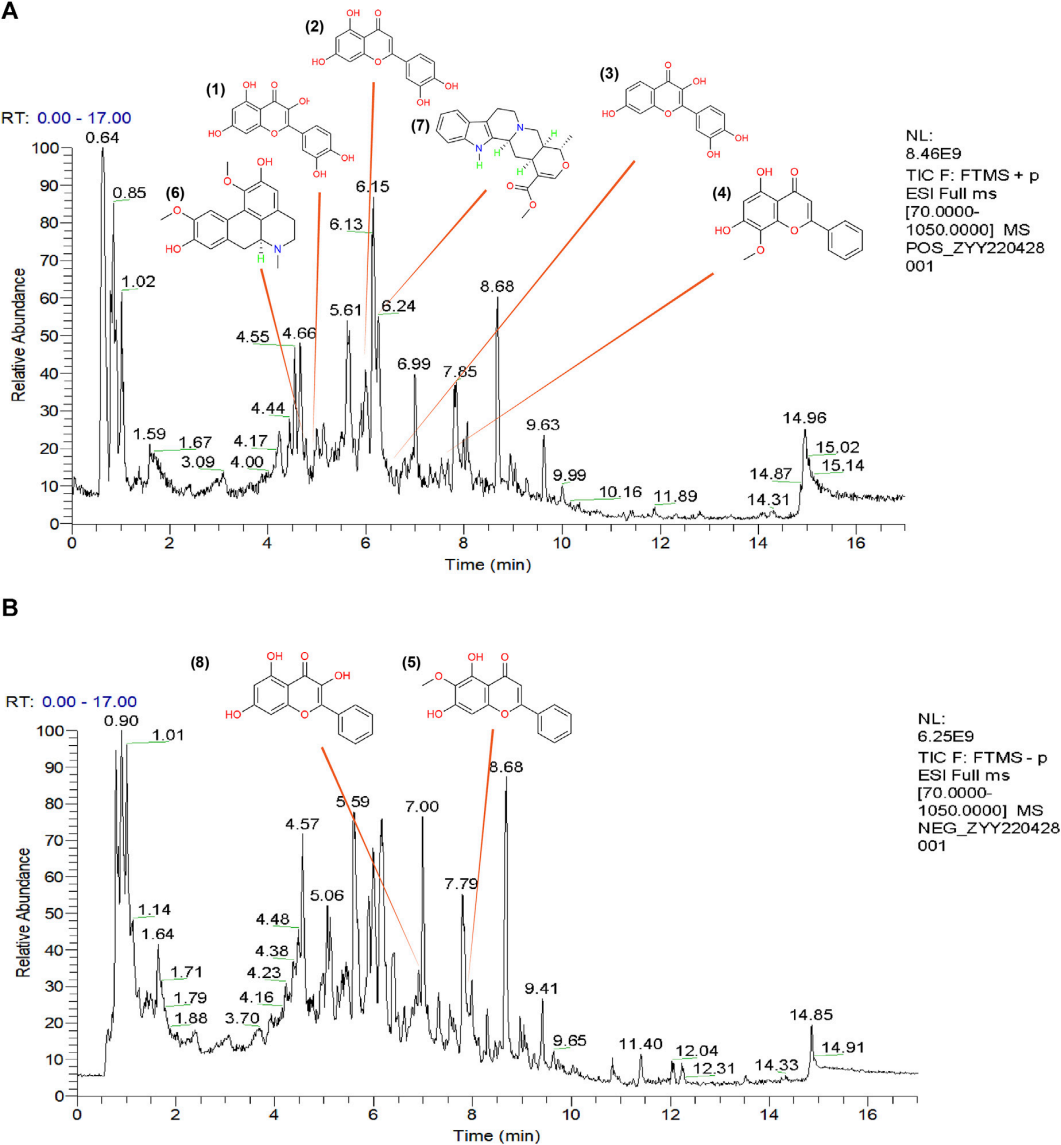
The total ion chromatograms (TICs) of TGD and chemical structure of the core components. **(A)** TIC of TGD in negative ion mode. **(B)** TIC of TGD in positive ion mode. 1) quercetin, 2) luteolin, 3) fisetin, 4) wogonin, 5) oroxylin A, 6) boldine, 7) tetrahydroalstonine, 8) galangin.

To further demonstrate the reliability of the results of network pharmacological prediction, and to determine the core components and targets of TGD in the treatment of alopecia, a second-dimensional network was constructed using bioactive components identified by UHPLC-MS/MS based on OB ≥ 30% and DL ≥ 18%. UHPLC-MS/MS identified altogether 251 bioactive components in TGD; their chemical compositions are listed in Supplementary Table S4 and the total positive and negative ion chromatograms are shown in Figures 3A, B. We then screened 39 bioactive compounds, and obtained 782 corresponding targets (after eliminating overlapping targets) in the TCMSP and Swiss target prediction databases (Supplementary Table S5).

**FIGURE 4:**
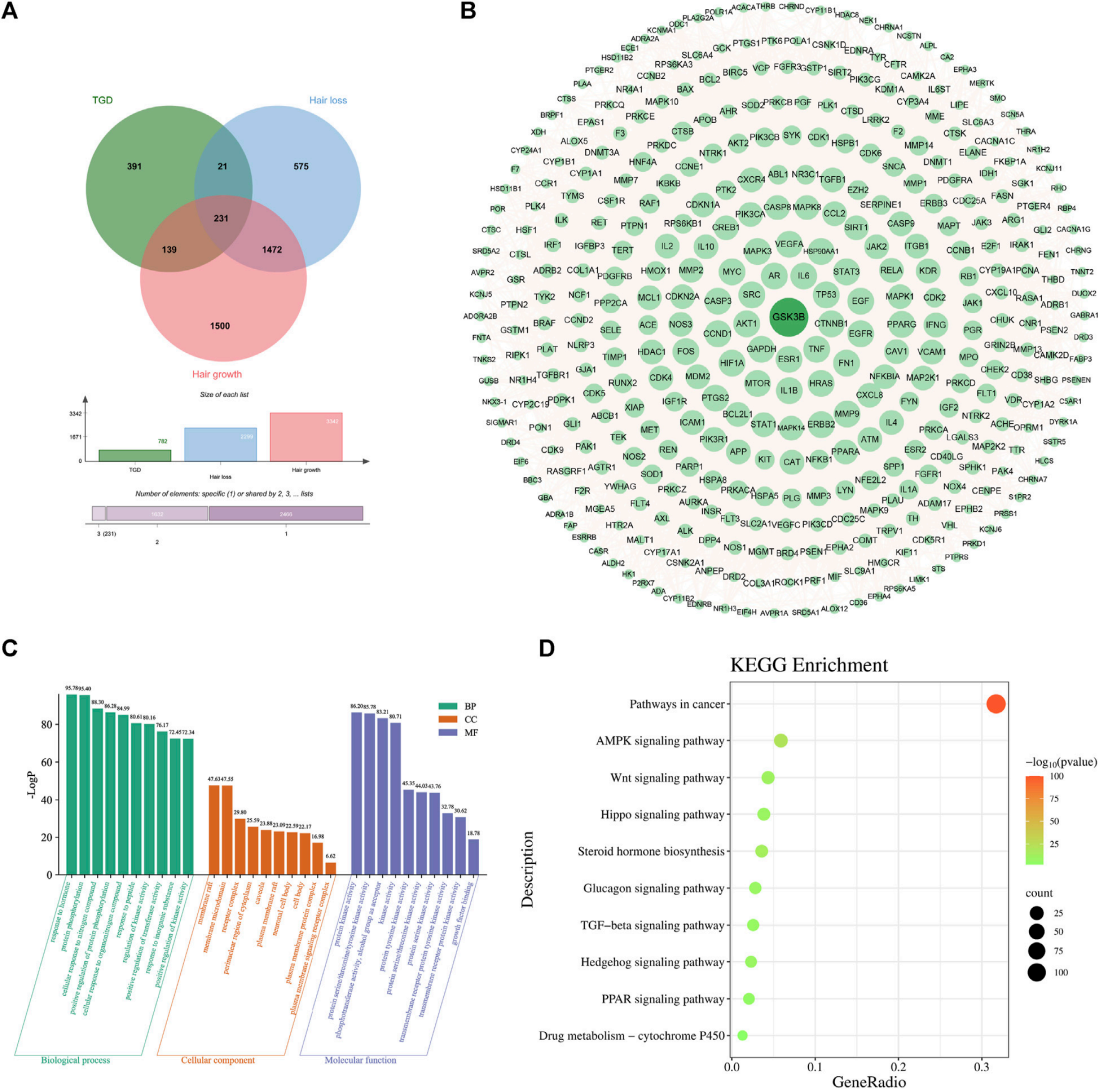
Second-dimensional network constructed by bioactive components detected from TGD in alopecia. **(A)** Venn diagram of diseases and bioactive compounds cross-targets. **(B)** Protein-protein interaction network. **(C)** The top 10 significantly enriched of BP, CC, and MF of GO analysis. The *X*-axis represents the pathway enrichment in GO category. The *Y*-axis shows the significant enrichment counts. **(D)** The KEGG pathway enrichment analysis of key targets.

The 782 targets and 3938 alopecia-associated targets identified in section 4.2 are represented on a Venn diagram (Figure 4A). A total of 391 overlapping genes were found to be targets of TGD in the treatment of alopecia, and were displayed in the PPI network diagram (Figure 4B). This network comprises 389 nodes and 11,063 edges, of which PDE1C and UTSR2 were not corelated with other nodes. Similarly, GSK3β, which is related to hair follicle development, were included in the target list as they exhibited degrees above the mean. GSK3β is ranked 40th in the PPI network based on degree.

GO analysis indicated that TGD treatment of hair loss is associated with responses to hormones, protein phosphorylation, and positive regulation of transferase activity (Figure 4C). Results of KEGG enrichment analysis verified that the effect of TGD on hair growth promotion is related to the Wnt signaling pathway (Figure 4D).

**FIGURE 5:**
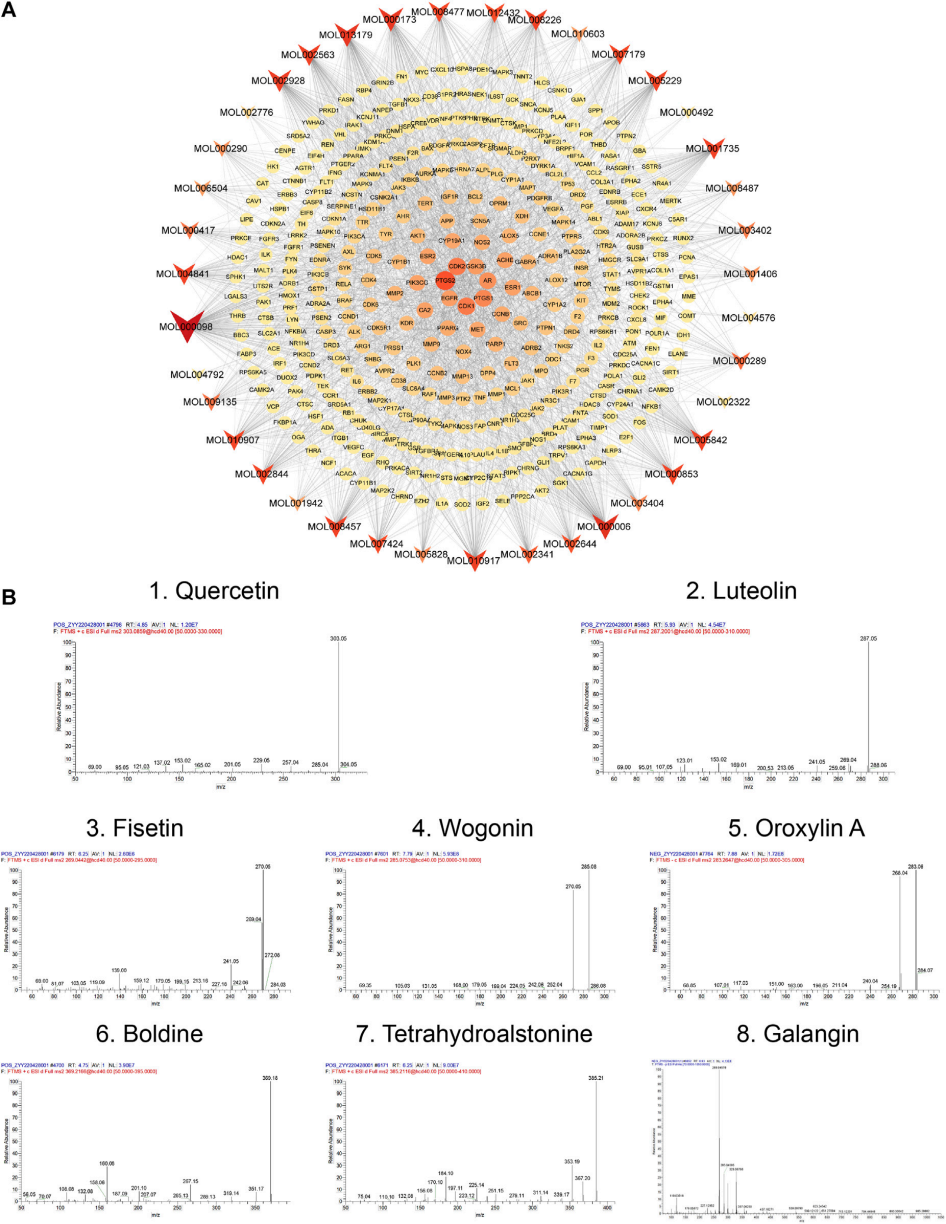
Network and Mass spectra analysis. **(A)** Bioactive component-target network. The ellipse nodes represent the overlapping gene targets between the alopecia disease and TGD. The triangle nodes are composed of bioactive components in TGD. The size and color depth of node were correlated with degree-value. **(B)** Mass spectra of the core components in TGD.

Subsequently, a bioactive component-target network was constructed using 39 bioactive compounds and 391 overlapping genes (Figure 5A). The network comprised 1988 edges and 430 nodes, and clearly illustrates that the therapeutic effect of TGD on alopecia is exerted by multiple components acting on multiple targets. The eight most prominent components were identified based on degree value, namely quercetin, luteolin, fisetin, wogonin, oroxylin A, boldine, tetrahydroalstonine, and galangin, with degree values of 165, 102, 90, 88, 77, 76, 71, 71, respectively. Their Mass spectra are shown in Figure 5B. The magnitude of the degree indicated importance of compounds or targets in the entire network. The results indicated that quercetin was likely the most important bioactive component associated with TGD alopecia treatment. In addition, the top eight targets were PTGS2, CDK2, CDK1, PTGS1, AR, GSK3β, EGFR, ESR1 with 49-degree, 36-degree, 36-degree, 32-degree, 29-degree, 26-degree, 25-degree, 24-degree, respectively.

### 3.4 Molecular docking visualization of TGD bioactive compounds and targets

**FIGURE 6:**
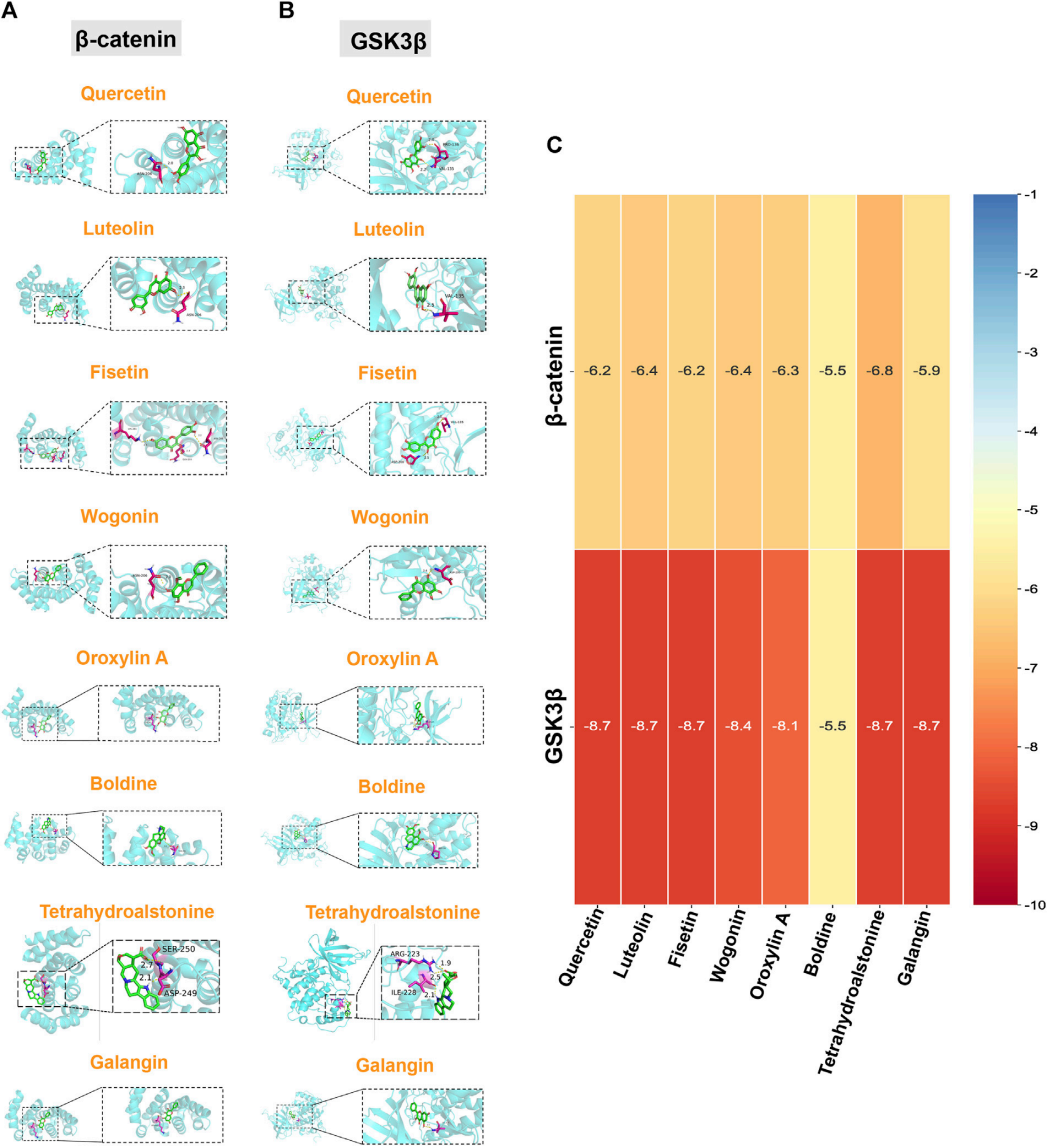
Molecular docking of core components and Wnt/β-catenin-related targets. Quercetin, luteolin, fisetin, wogonin, oroxylin A, boldine, tetrahydroalstonine, and galangin with **(A)** β-catenin, and **(B)** GSK3β. **(C)** Cluster heatmap of docking results. Numerical value indicated the binding energy ((kcal/mol).

To further verify and evaluate the effective binding of the identified bioactive compounds with their predicted targets, we screened eight components as main bioactive components of TGD by bioactive component-target network analysis. More specifically, we docked the top eight components (quercetin, luteolin, fisetin, wogonin, oroxylin A, boldine, tetrahydroalstonine, and galangin) with two targets in the Wnt/β-catenin pathway (GSK3β and β-catenin), respectively. A binding energy of < -5 kcal/mol was set as the threshold, the magnitude of which indicates the stability and strength of the binding. The binding energy of all ligands was greater than the threshold (Figures 6A–C), indicating that all ligands possessed strong binding ability with their receptors. The binding energy of tetrahydroalstonine was the highest with β-catenin, at -6.8 kcal/mol (Figure 6C); whereas quercetin, luteolin, fisetin tetrahydroalstonine, and galangin displayed the highest binding energy with GSK3β, at -8.7 kcal/mol (Figure 6C).

### 3.5 Effect of TGD on hair cycle in C57BL/6J mice

**FIGURE 7:**
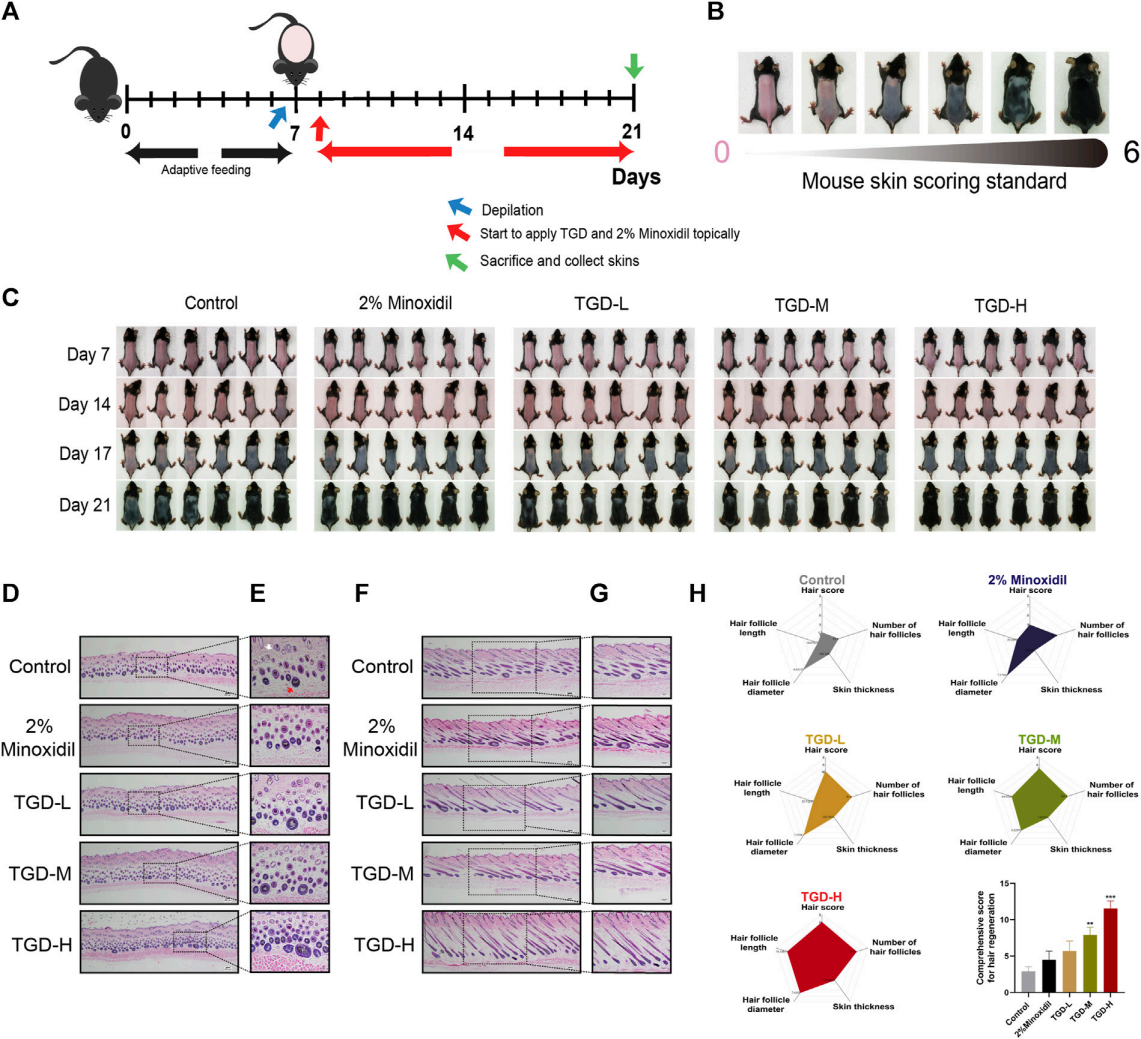
TGD promoted hair follicle development in C57BL/6J mice. **(A)** Timeline of TGD-administrated mice. **(B)** Mouse skin score standard. **(C)** The changes of dorsal skin in C57BL/6J mice treated with TGD-L (40 mg/kg), TGD-M (200 mg/kg) and TGD-H (400 mg/kg) at the indicated times. **(D–G)** H&E staining of full-thickness skin, **(D,F)** with magnification ×40. **(E)** with magnification × 200. **(G)** with magnification ×100. **(D,E)** Representative microimages of skin cross sections and **(F,G)** skin longitudinal sections. White arrow point to telogen follicles. Red arrow point to anagen follicle. **(H)** Comprehensive score for hair regeneration (*n* = 5). Data were presented as mean ± standard error of the mean (SEM). **p < 0.01, ***p < 0.001 vs. the control group.

To determine the hair-promoting effect of TGD *in vivo*, we depilated seven-week-old C57BL/6J mice so that they were at the same hair growth phase, and then topically administered 2% minoxidil or TGD. The timeline of TGD-administrated mice and mouse skin score standard were shown in Figures 7A, B respectively. As expected, treatment with 2% minoxidil promoted hair growth (Figure 7C). By day 14, all mice in the TGD-H group, and most mice in the TGD-L and TGD-M groups, had entered anagen, while those in the control group remained in the telogen phase. By day 17, all mice in all treatment groups had entered the anagen phase in a dose-dependent manner. Therefore, the preliminary results indicated that TGD significantly promoted hair growth. That is, TGD shortened telogen and induced the anagen phase of hair growth earlier in C57BL/6J mice.

To further illustrate this point, histological analysis revealed that the number of hair follicles was higher in the TGD and 2% minoxidil groups than in the control group; in the treatment groups, most hair follicles were in anagen (Figures 7D, E) and the thickness of full-thickness skin was increased (Figures 7F, G). We visualized the data through radar charts (Figure 7H), using hair score, number of hair follicles, skin thickness, hair follicle diameter, and hair follicle length as parameters. Statistical results showed that both the TGD-M and TGD-H groups significantly induced hair follicle regeneration and promoted hair growth (*p* < 0.01 and *p* < 0.001, respectively).

### 3.6 Effect of TGD on hair shaft elongation and DPCs proliferation *ex vivo*

**FIGURE 8:**
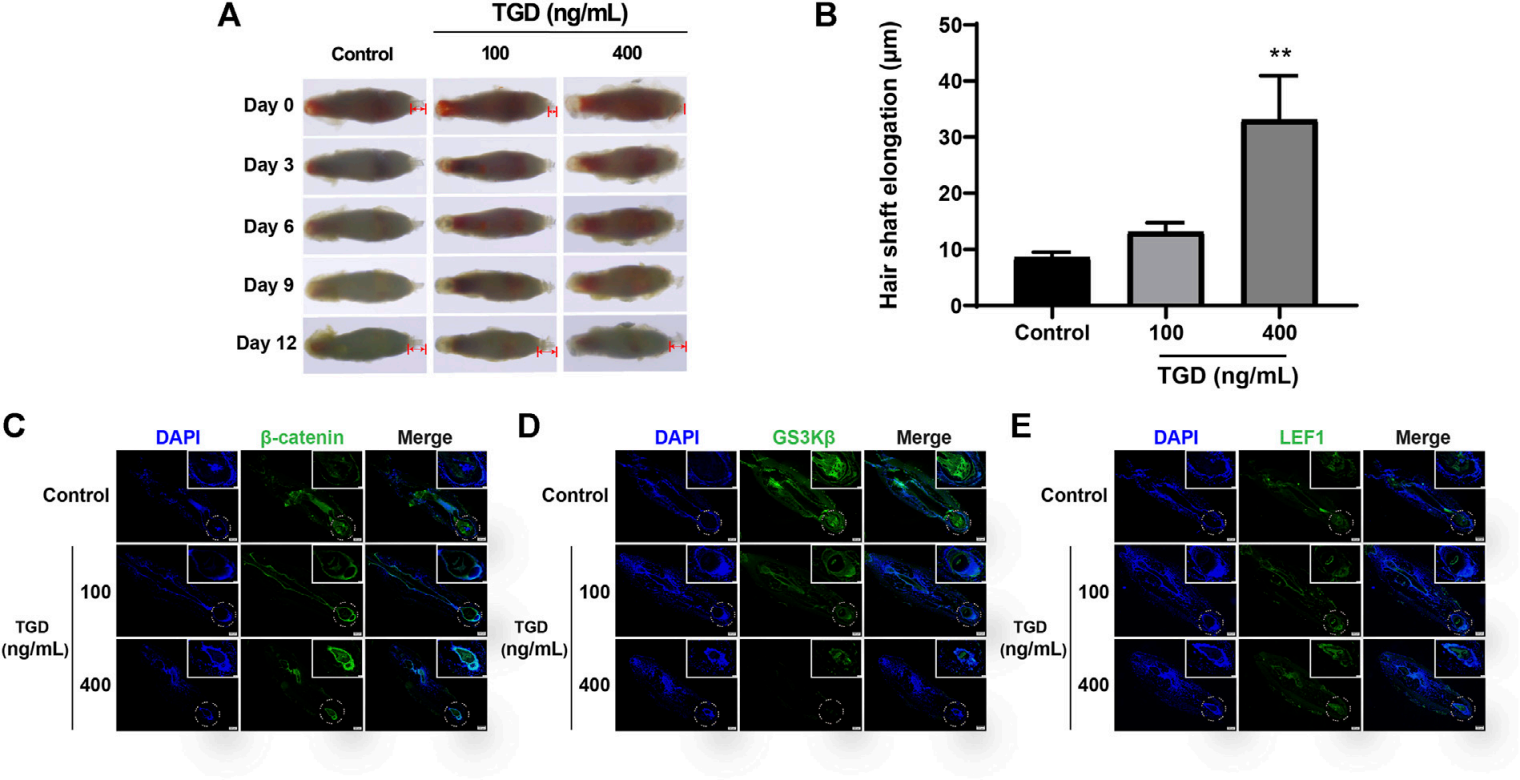
TGD promoted hair shaft elongation in rat vibrissa follicles. **(A)** Photographs of rat vibrissa follicles cultured with TGD for 0, 3, 6, 9, 12 days; The double arrow area represents the measured hair shaft length. **(B)** Changes in length of hair shafts in the vibrissa follicles treated with TGD for 12 days. Data are presented as mean ± SEM (*n* = 6). **p < 0.01 compared with the control group. **(C–E)** The representative images of immunofluorescence staining in rat vibrissa follicles for β-catenin, GSK3β, LEF1 (×40), picture of magnification ×200 in white box.

We evaluated the effect of TGD in isolated rat vibrissa follicle tissues. At day 12, the hair shafts of rats that had received a dose of 400 ng/ml TGD were elongated compared with those of the control group (*p <* 0.01, Figures 8A, B). Furthermore, TGD promoted the proliferation of DPCs in a dose-dependent manner, with a dosage of 400 ng/ml being the most effective (*p <* 0.01, Supplementary Figure S1).

### 3.7 Effect of TGD on the expression of core proteins in hair follicles and DPCs

**FIGURE 9:**
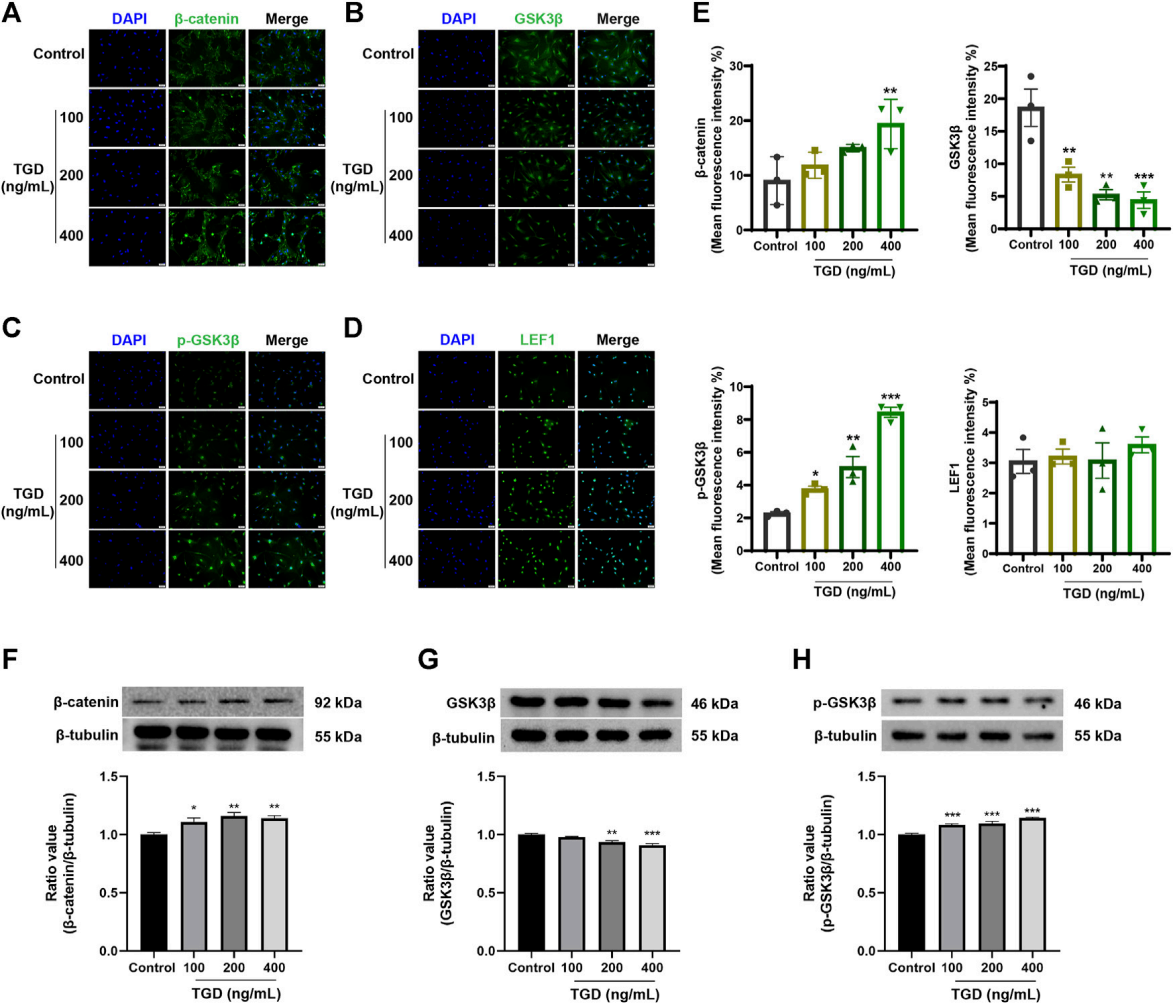
**(A–E)** The representative images and statistical graph of β-catenin, GSK3β, p-GSK3β and LEF1 in DPCs (×200), *n* = 3 per group, *p < 0.05, **p < 0.01, and ***p < 0.001 vs. the control group. **(F–H)** Western blot analysis of Wnt/β-catenin signaling pathways related protein expression in DPCs, *n* = 4 per group, *p < 0.05, **p < 0.01, and ***p < 0.001 vs. the control group.

The expression of core proteins related to the Wnt/β-catenin signaling pathway was monitored by immunofluorescence staining and western blot. Immunofluorescence results demonstrated that the abundance of β-catenin and LEF1 increased while levels of GSK3β decreased after TGD intervention in rat vibrissa follicles and DPCs (Figures 8C–E, Figures 9A, B–D). Synchronously, DPCs treated with TGD exhibited more significant expression of p-GSK3β than those in the control group (Figure 9C). Furthermore, we found that TGD treatment not only promoted the nuclear translocation of β-catenin in DPCs but also increased its expression in the cell membrane, with a trend toward a dose-dependent increase; the effect of TGD was significant at a 400 ng/ml dosage (*p <* 0.01, Figure 9E). Western blotting further confirmed that, compared with untreated controls, TGD upregulated β-catenin and p-GSK3β expression while downregulating that of GSK3β in DPCs, particularly in the TGD-H group compared with that of the control group (*p <* 0.05, *p <* 0.01, and *p <* 0.001; Figures 9F–H). These results suggest that TGD not only increased the expression of β-catenin but also inactivated GSK3β by promoting GSK3β phosphorylation, thereby reducing damage to β-catenin, which translocated into, and accumulated in, the nucleus to activate the Wnt/β-catenin signaling pathway.

### 3.8 Effect of TGD on proteins related to the Wnt/β-catenin signaling pathway *in vitro*

**FIGURE 10:**
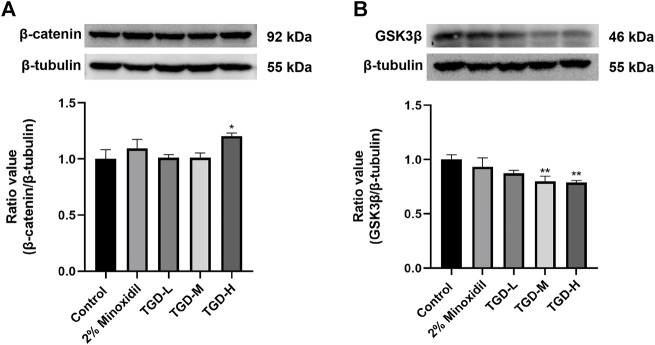
**(A,B)** Experimental validation of the Wnt/β-catenin signaling pathways *in vivo* (*n* = 5). The data are expressed as the mean ± SEM. *p < 0.05, **p < 0.01, and ***p < 0.001 vs. the control group.

To further confirm our findings, we evaluated the effects of TGD on GSK3β and β-catenin expression levels in C57BL/6J mice tissues. Western blotting confirmed that, compared with untreated controls, the β-catenin level of the TGD group was higher than that of the untreated-group (*p <* 0.05; Figure 10A). Moreover, TGD downregulated GSK3β expression in C57BL/6J mice, particularly in the TGD-H group, which exhibited a significant hair promotion effect (*p <* 0.01; Figure 10B).

## 4 Discussion and conclusion

TCM contains a variety of biologically active compounds and has been widely used in the treatment of various diseases due to its beneficial therapeutic effects, few side effects, and its multi-component and multi-target synergistic value (Bu et al., 2020; Luan et al., 2020; Wang et al., 2021). However, assessing the efficacy of compound herbal therapies can be challenging. Emerging network pharmacology is an ideal tool for the study of the mechanisms underlying TCM treatment, which is helpful to promote the development of TCM preparations (Sucher, 2013; Nogales et al., 2022). Network pharmacology combines computer science with biological information, constructing networks using algorithms, theoretical data, and experimental data; this allows the purposeful and directional investigation of the therapeutic mechanisms of TCM on a molecular and biological level (Zhao et al., 2019). In our study, we expanded on conventional network pharmacology to construct a dual-dimensional network for analyzing the mechanism of TGD treatment of alopecia, using both theoretical and experimental data. This method seeks to evaluate the authenticity and reliability of the action targets of TGD for alopecia. A total of 39 bioactive components of TGD were detected by UHPLC-MS/MS. Subsequently, a bioactive component-target network was constructed to predict the protein targets and bioactive components of TGD for treating hair loss. Comprehensive GO, KEGG, and molecular docking analyses revealed the binding capacity between the target proteins and bioactive components, as well as the potential signaling pathway underlying the TGD anti-alopecia effects. After analysis of the networks, we used a murine model of alopecia confirmed the efficacy of TGD *in vivo* in promoting hair growth. Finally, Western blot and immunofluorescence analysis were performed to verify the result of network pharmacologic prediction, elaborate *in vitro* and *in vivo* the mechanisms of TGD against alopecia.

A total of 251 active ingredients were detected in the TGD extracts using UHPLC-MS/MS, of which 39 bioactive components satisfied the condition of OB ≥ 30% and DL ≥ 0.18. According to the analysis of bioactive component-target network and references, quercetin, luteolin, fisetin, wogonin, oroxylin A, boldine, tetrahydroalstonine, and galangin were likely the most important bioactive compounds associated with the anti-alopecia activity of TGD, which relative contents in TGD extract were 0.16%, 0.35%, 0.002%, 2.03%, 1.17%, 0.12%, 0.45% and 0.06% respectively (Supplementary Table S6). Among them, Quercetin, luteolin, wogonin, oroxylin A and tetrahydroalstonine have high relative contents in TGD extract. There is a good correlation between the content of compound and its importance. Furthermore, one of the causes of hair loss is oxidative stress due to increased free radicals in the hair follicle (Ablon, 2021). Quercetin has a significant antioxidative role in many diseases (Cebecioglu et al., 2019; Tang et al., 2019; Zou et al., 2021). Quercetin also can stimulate the proliferation of human dermal papilla cells by activating the MAPK/CREB signaling pathway, thereby promoting the production of growth factors, and stimulating the elongation of human hair follicles (Kim et al., 2020). Studies have shown that quercetin can promote hair growth in C3H/HeJ mice and stimulate the proliferation of epithelial cells, which is considered as a potential hair protectant (Towatari et al., 2002; Wikramanayake et al., 2012; Dario et al., 2020). Luteolin has been shown to significantly promote hair regeneration *in vitro* (Shin et al., 2018; Taguchi et al., 2019). The anti-alopecia activities of fisetin have been validated *in vivo* and *in vitro* studies, which might be achieved by enhancing telomerase reverse transcriptase (TERT) levels (Kubo et al., 2020; Ogawa et al., 2021). Moreover, Quercetin, luteolin and fisetin also have significant anti-androgen effect (Khan et al., 2008; Das et al., 2019; Wu et al., 2021). It is noteworthy that quercetin, luteolin, fisetin, and wogonin are flavonoids, which have a significant effect on protecting hair follicles, and constitute a class of natural medicines with great potential to prevent hair loss (Bassino et al., 2020). Wogonin and oroxylin A existed in Scutellaria baicalensis Georgi, and tetrahydroalstonine is unique ingredient in Uncaria rhynchophylla (Miq.) Miq. Ex Havil. Studies have shown that extract of Scutellaria baicalensis Georgi can effectively inhibit the expression of androgen receptor, thus inhibiting hair loss (Kim et al., 2014). Oroxylin A have been shown that could reduce the expression of inflammatory factors in human dermal fibroblasts thereby inhibiting the damage to human dermal fibroblasts (Nakajima et al., 2001). As for wogonin, oroxylin A and tetrahydroalstonine these compounds get more attention on regulating hormone levels activity (Roquebert and Demichel, 1984; Chi and Kim, 2005; Wang et al., 2013). Galangin can promote hair darkening in mice *in vitro*, but the specific mechanism needs further study (Huo et al., 2014). Collectively, all of which indicate that these ingredients have potent research value in promoting hair growth activity, and TGD is a TCM preparation with positive therapeutic anti-alopecia effects.

The PPI results identified GSK3β as potential targets for TGD treatment of alopecia. GSK3β is defined as a serine-threonine kinase involved in cell motility and development as well as the regulation of glucose homeostasis. Moreover, GSK3β protein has a key role in the Wnt/β-catenin signaling pathway. GSK3β regulates this pathway by governing the stability and function of β-catenin (Gao and Chen, 2010), which is a substrate of GSK3β and a core protein of the pathway. Furthermore, we found that AR and ESR1 proteins related to hair growth are also included. GSK3β can inhibit the activity of AR (Salas et al., 2004), which is a member of the nuclear receptor superfamily that enters the nucleus to regulate the expression of downstream genes after binding to testosterone or dihydrotestosterone (Li et al., 2019). Dysregulation of intracellular androgen levels can cause hair loss, and ARs mediate the circulation of androgen steroid hormones in cells (Lai et al., 2012). More specifically, ARs phosphorylate β-catenin by activating GSK3β in DPCs; phosphorylated β-catenin is degraded by proteasomes, thereby inhibiting activation of the Wnt/β-catenin pathway (Leirós et al., 2012). Meanwhile, ESR1 is an estradiol receptor that induces β-catenin stabilization by promoting the phosphorylation of GSK3β at serine 9 (Varea et al., 2009). In addition, ESR1 can regulate β-catenin/TCF-mediated transcription, possibly regulating transcription of the Wnt protein, thereby triggering activation of the Wnt/β-catenin pathway (Varea et al., 2010). Finally, we validated binding affinity between eight key bioactive components and core targets (GSK3β, β-catenin) utilizing molecular docking to provide further support for the role of TGD in treating alopecia. Molecular docking outcomes manifested that the eight components had strong intermolecular interactive forces with GSK3β and β-catenin protein.

GO and KEGG enrichment analysis confirmed that the role of TGD in hair follicle regeneration was related with Wnt pathway, hormone response, and regulation of cell migration and movement. Wnt/β-catenin signaling pathway plays a crucial role in embryonic hair follicle morphogenesis and is also one of the important pathways affecting hair follicle development and regeneration during the hair cycle. (Rishikaysh et al., 2014; Kim and Garza, 2017). This signaling pathway promotes hair follicle regeneration by regulating hair follicle cycle. Research shows that Dickkopf-1, as an inhibitor of Wnt/β-catenin signaling pathway, has the effect of promoting hair follicle transition from anagen to telogen phase (Kwack et al., 2012). In addition, inactivation of β-catenin in the dermal papilla also leads to enter telogen phase in advance of hair follicles (Enshell-Seijffers et al., 2010). β-catenin is activated and transferred to the nucleus for expression, which is a key signal mediating transcription and activating Wnt/β-catenin signaling pathway (Logan and Nusse, 2004; Shimomura and Christiano, 2010). GSK3β forms a complex with the scaffolding protein Axin (Axin), casein kinase 1α (CK1), and adenomatous polyposis coli (APC) without Wnt ligand, thereby disrupting the signaling pathway by phosphorylating the Thr41/Ser37/Ser33 site of β-catenin, leading to its proteasomal degradation (Nusse and Clevers, 2017). The transcriptional function of β-catenin is mainly regulated by Axin/GSK-3β/APC complex (Jin et al., 2021). When the Wnt/β-catenin pathway is activated, the complex is disassembled, resulting in translocation of β-catenin from the cytoplasm to the nucleus (Angers and Moon, 2009).

**FIGURE 11:**
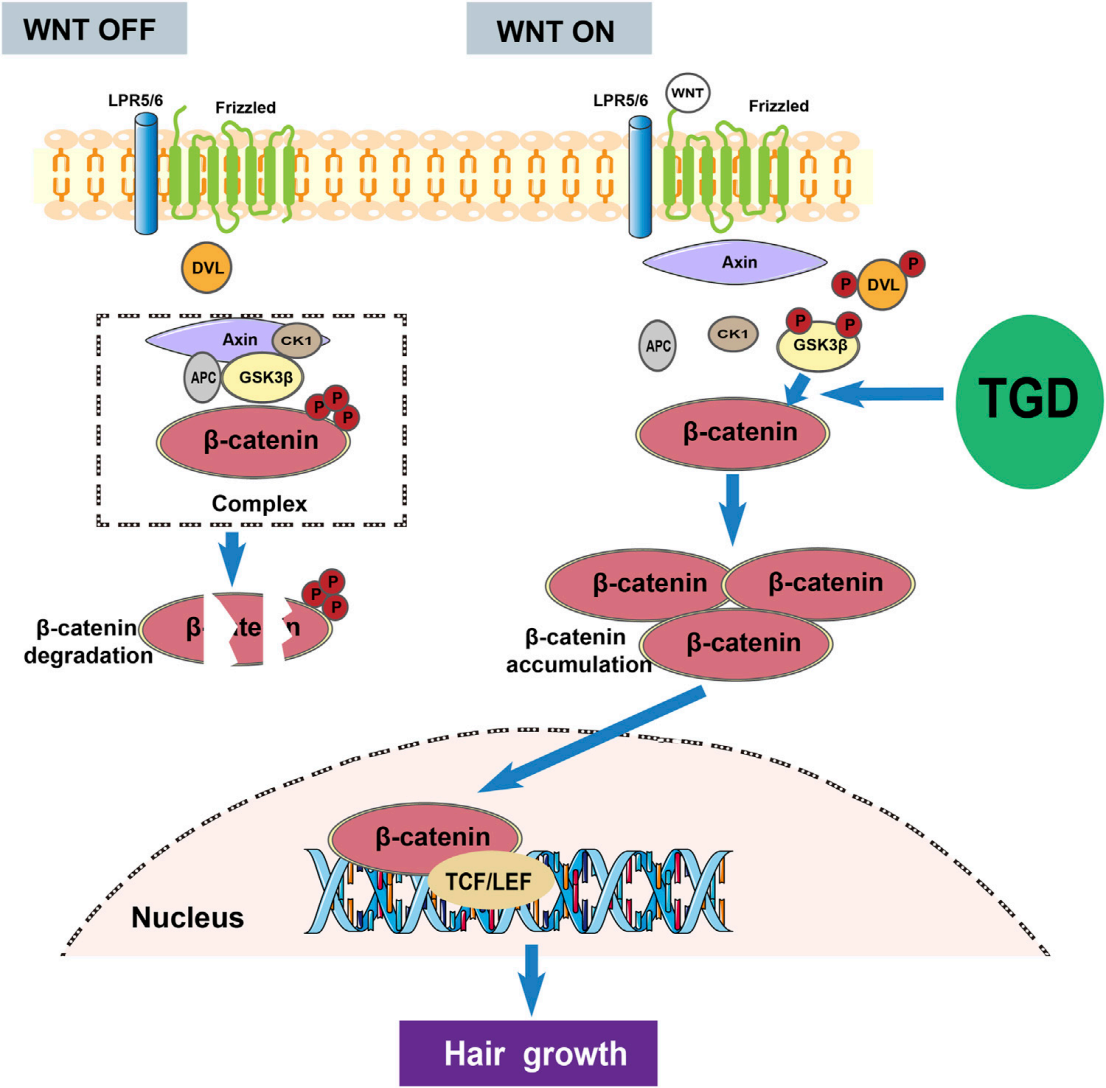
The overall mechanism diagram involved in the activation effect of TGD on hair growth.

We also evaluated the efficacy of TGD in hair follicle regeneration in C57BL/6J mice. Results showed that the mice entered the telogen after depilation, however, following administration of TGD, they entered anagen at an accelerated rate. Hence, TGD effectively promoted hair growth in C57BL/6J mice. Additionally, treatment of rat vibrissa follicle and DPCs with TGD caused accumulation of β-catenin and LEF1 in the nuclei. Furthermore, TGD treatment significantly stimulated the expression of β-catenin and p-GSK3β, while decreasing that of GSK3β in DPCs and C57BL/6J mice. Hence, TGD may promote the phosphorylation of GSK3β in the cytoplasm, which in turn inhibits β-catenin phosphorylation by GSK3β, ultimately leading to accumulation of β-catenin in the cell nuclei and activation of the Wnt/β-catenin pathway (Figure 11). These results confirm the prediction of network pharmacology and molecular docking analyses, providing experimental reference for the mechanism employed by TGD against alopecia.

Collectively, this study provides several new directions for the development of subsequent therapeutics. For example, ESR1 and AR were identified as targets of TGD for the treatment of hair loss; we postulated that TGD may alleviate hair loss by promoting the expression of estrogens, thus counteracting the increase in androgen levels; however, this conjecture requires further confirmation. Moreover, we have shown that dual-dimensional network pharmacology is an effective strategy that builds on basic network pharmacology for a more accurate investigation into the possible therapeutic mechanisms and compounds of drugs. This will be increasingly important as multi-target therapies become more common as a strategy for the treatment of hair loss.

In conclusion, we show that TGD has positive therapeutic effects on hair growth and demonstrate the reliability of network pharmacology in the study of drug mechanisms. Hence, this study lays the foundation for the development of new therapeutics based on TCM.

## Data Availability

The datasets presented in this study can be found in online repositories. The names of the repository/repositories and accession number(s) can be found in the article/Supplementary Material.
