# Supplementary material for: Stimulation of hair growth by Tianma Gouteng decoction: Identifying mechanisms based on chemical analysis, systems biology approach, and experimental evaluation
Source: Front Pharmacol. 2022 Dec 16;13:1073392. doi: 10.3389/fphar.2022.1073392 (PMC9802907; doi:10.3389/fphar.2022.1073392)
Supplement: Supplementary file 1 [file DataSheet1.zip › Supplementary date/Supplementary Figure S1.docx]

## Supplementary Figures S1


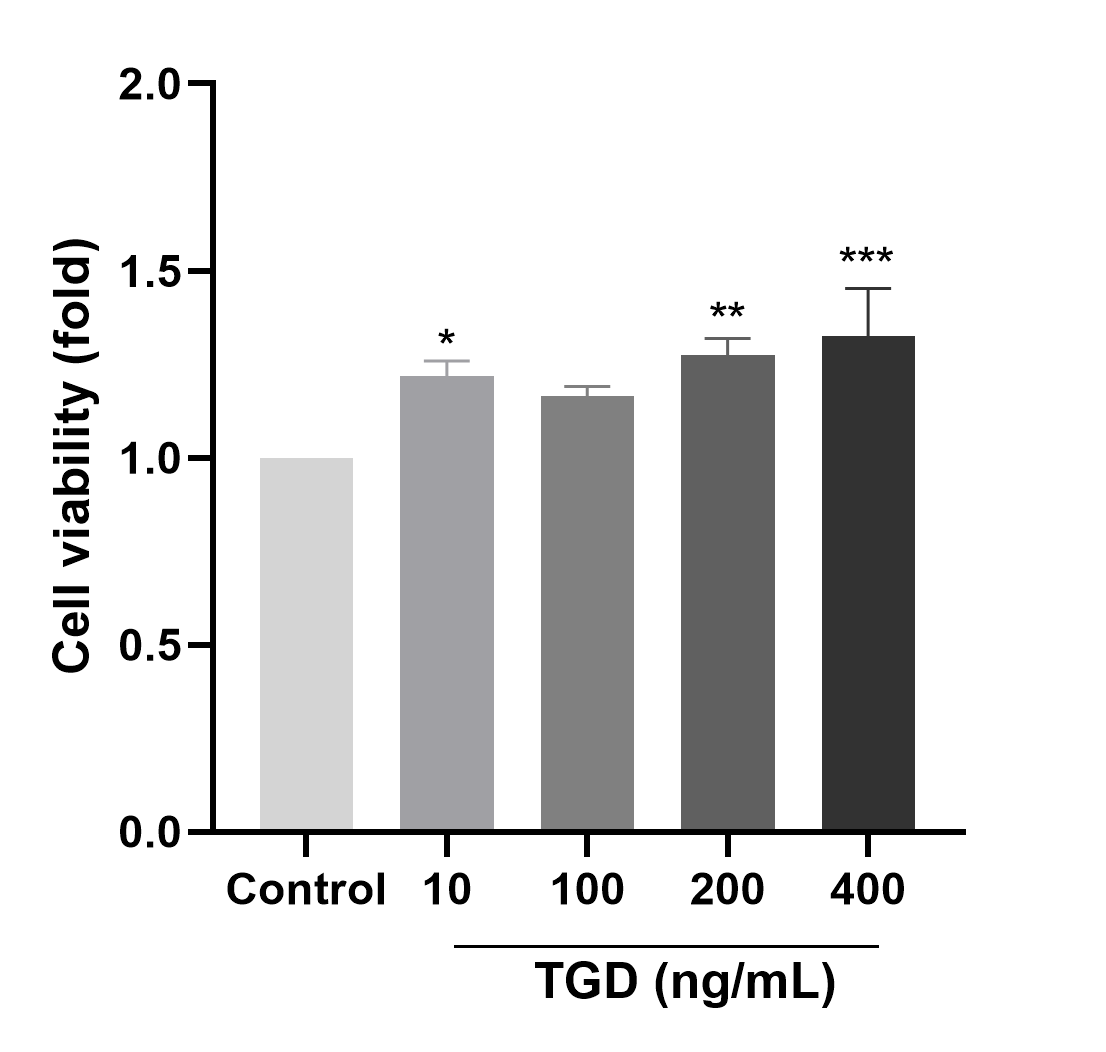


**Supplementary Figure S1.** The detailed results of cell viability assay. **P* < 0.05, ***P* < 0.01, ****P* < 0.001 vs the control group.
